# The Impact of Various Surface Treatments on Adhesion Between Co–Cr Alloy and Acrylic Teeth

**DOI:** 10.1155/ijod/2199689

**Published:** 2026-02-05

**Authors:** Punnita Chotcomwongse, Pongsakorn Apinsathanon, Basel Mahardawi, Palawat Laoharungpisit, Pheeradej Na Nan, Napapa Aimjirakul

**Affiliations:** ^1^ Department of Conservative Dentistry and Prosthodontics, Faculty of Dentistry, Srinakharinwirot University, 114 Soi Sukhumvit 23, Khlong Toei Nuea, Watthana, Bangkok, 10110, Thailand, swu.ac.th; ^2^ Department of Prosthodontics, Faculty of Dentistry, Chulalongkorn University, Bangkok, Thailand, chula.ac.th; ^3^ Department of Oral and Maxillofacial Surgery, Faculty of Dentistry, Chulalongkorn University, Bangkok, Thailand, chula.ac.th; ^4^ Department of General Dentistry, Faculty of Dentistry, Srinakharinwirot University, Bangkok, Thailand, swu.ac.th; ^5^ National Cyber Security Agency, Bangkok, Thailand

**Keywords:** adhesive, artificial teeth, dental cements, polymethyl methacrylate, surface modification

## Abstract

**Objective:**

This study aimed to assess the shear bond strength (SBS) between cobalt–chromium (Co–Cr) alloy and acrylic resin teeth using various surface conditioning methods and to investigate the failure modes associated with these methods.

**Materials and Methods:**

A total of 53 Co–Cr specimens were fabricated and randomly assigned to five groups (*n* = 10 per group) based on the type of surface treatment: (1) polymethyl methacrylate (PMMA; control group), (2) Clearfil Ceramic Primer Plus + PMMA, (3) M&C Primer + PMMA, (4) Super‐Bond Universal, and (5) M&C Primer + Super‐Bond Universal. Each specimen was subsequently bonded to an acrylic resin tooth. Three specimens from both the control and primer‐treated groups were reserved for scanning electron microscopy (SEM) analysis. All samples were stored in distilled water at 37 ± 1°C for 24 h before SBS evaluation with a universal testing machine.

**Statistical Analysis:**

Statistical analysis was performed using one‐way analysis of variance (ANOVA) followed by Tukey’s HSD post hoc test.

**Results:**

A statistically significant difference in bond strength was observed among the experimental groups (*p* < 0.05), with the Super‐Bond Universal group exhibiting the highest bond strength (8.01 ± 1.05 megapascals [MPa]), compared to the control group (3.07 ± 0.48 MPa). The control group and the PMMA + M&C Primer group exhibited predominantly adhesive failures, while the other groups showed a combination of adhesive and mixed failures.

**Conclusion:**

Super‐Bond Universal without primer achieved the highest bond strength, and the application of primers prior to PMMA or resin cement placement significantly improved bonding between Co–Cr alloys and acrylic resin teeth, suggesting that appropriate surface conditioning can enhance denture repair outcomes.

## 1. Introduction

Removable partial dentures (RPDs) are widely used due to their cost‐effectiveness, noninvasive nature, and ease of fabrication. Traditionally, metal RPDs were fabricated from cobalt–chromium (Co–Cr) alloys for the metal framework, owing to their optimal balance of strength, corrosion resistance, and cost‐effectiveness of the metal [1]. Polymethyl methacrylate (PMMA) resin is predominantly used for denture bases and artificial teeth. Its attachment to metal relies mainly on mechanical interlocking, which may become insufficient over time, particularly under masticatory forces, leading to debonding or interfacial fracture [2].

Debonding of acrylic resin teeth from metal denture bases remains one of the most frequently reported mechanical complications, with incidence rates reaching up to 33% [3]. This issue is particularly prevalent in the anterior region, where limited available space is often associated with malocclusions such as a deep bite [4]. Other contributing factors include wax contamination during processing [5], a mismatch in thermal expansion coefficients between the acrylic resin and metal alloys [6], and insufficient monomer infiltration [7].

When an artificial tooth becomes dislodged from a denture base, management typically involves either the fabrication of a new prosthesis or the repair of the existing denture. Due to its cost‐effectiveness and shorter chairside time, denture repair is generally regarded as a practical alternative to complete prosthesis replacement. Conventionally, this issue is addressed by repositioning the dislodged tooth and securing it to the metal framework using mechanical retention techniques, such as retentive grooves, grinding, and air abrasion [8–11].

Nonetheless, various chemical agents have been developed to enhance bonding and achieve optimal bond strength between resin materials and metal surfaces. Previous studies have demonstrated that the application of primers containing functional monomers such as 3‐(trimethoxysilyl)propyl methacrylate (3‐MPS), 10‐methacryloyloxydecyl dihydrogen phosphate (10‐MDP), and 4‐methacryloxyethyl trimellitate anhydride (4‐META) can enhance chemical bonding at the interface between Co–Cr alloys and acrylic resin [12–14]. Although 3‐MPS is primarily designed for silica‐based ceramics, it can bond to Co–Cr alloys via hydroxyl groups on the oxidized metal surface, with its silanol group promoting adhesion and its methacrylate group enabling copolymerization with PMMA [15]. Similarly, 10‐MDP and 4‐META contain functional groups, phosphate and carboxylic, respectively, that chemically interact with the metal oxide layer, while their methacrylate groups enable covalent bonding with acrylic resins, thereby enhancing overall bond durability [16–18].

A variety of primers are currently available for clinical application. However, evidence regarding the performance of Clearfil Ceramic Primer Plus and M&C Primer, both of which incorporate these monomers, as well as their combined use with resin cements, remains relatively limited. Further investigation of these materials is important to clarify their effectiveness in enhancing the bond strength between acrylic resin and metal frameworks, thereby supporting their clinical applicability.

Therefore, this study aimed to assess the shear bond strength (SBS) between Co–Cr alloy and acrylic resin teeth using various surface conditioning methods and to investigate the failure modes associated with these methods. The null hypothesis was that different surface conditioning methods would not significantly affect the SBS between Co–Cr alloy and acrylic resin teeth.

## 2. Materials and Methods

The sample size was determined using G∗Power 3.1.9.4. Means and standard deviations for SBS were obtained from a previous study by Kalra et al. [19]. Based on the calculation with an effect size of 0.716, a significance level (*α*) of 0.05, and a desired power of 0.95, the required sample size was 10 specimens per group.

A total of 53 rectangular printed‐resin specimens (dimensions: 10 × 10 × 2.5 mm^3^) were utilized as patterns for the fabrication of Co–Cr alloy specimens (Vitallium Alloy, Dentsply Prosthetics, York, USA). Each specimen was embedded in an epoxy resin block, leaving the surface exposed to facilitate subsequent bonding procedures. To remove surface contaminants and ensure uniformity, the exposed metal surfaces were polished using 600‐grit silicon carbide abrasive paper (TOA Abrasives, TOACP, Samut Prakan, Thailand) at 300 rpm for 20 s each (NANO‐1000S, Pace Technologies, Arizona, USA). Surface treatment was performed by air abrasion using 50 µm aluminum oxide (Al_2_O_3_) particles (white Al_2_O_3_, Zest Dental Solution, Berlin, Germany) for 10 s, maintaining a distance of 5 mm and an air pressure of 0.5 megapascals (MPa;Basic Eco Fine, Renfert GmBh, Baden‐Württemberg, Germany). Following air abrasion, the specimens were cleaned in distilled water for 10 min using an ultrasonic cleaner (SONOREX DIGITEC DT 100 H, Bandelin, Berlin, Germany) and then dried with an oil‐free compressed air system.

A total of 50 acrylic teeth (Yamahashi New Ace & FX, Yamahachi Dental MFG., CO., Aichi, Japan) were utilized. Each tooth was standardized by machining it into a cylindrical shape with a diameter of 6 mm and a height of 4 mm to ensure consistency across all bonding tests. An oriented polypropylene (OPP) tape with a thickness of 80 µm was trimmed to form a circular opening with a 6 mm circumference, designating the bonding area on the metal surface. The specimens were randomly divided into five groups, with 10 samples for each group, based on the type of surface treatment applied.

For primer preparation, M&C Primer (Sun Medical Co., Ltd., Shiga, Japan) was prepared by mixing equal volumes of Universal Primer Parts A and B in a 1:1 ratio, whereas Clearfil Ceramic Primer Plus (Kuraray Noritake Dental Inc., Tokyo, Japan) was applied by dispensing a single drop. In all primer‐treated groups, the primer was applied to the metal surface for 15 s and then air‐dried. For bonding with an autopolymerizing acrylic resin (PMMA), both the control and primer‐treated groups (Clearfil Ceramic Primer Plus and M&C Primer) underwent the bonding procedure using PMMA (Unifast Trad, GC Dental Products Corp., Aichi, Japan). The resin was prepared by mixing 1 g of powder with 0.5 mL of liquid. The components were thoroughly mixed for 10–15 s and subsequently applied to the bonding surface. The prepared cylindrical acrylic resin was placed on top of the PMMA. To maintain consistent bonding pressure, a glass slab was placed over the specimen, followed by the vertical application of a 500 g standard weight to ensure uniform contact and stability during polymerization. The specimens were left undisturbed until fully set.

Moreover, scanning electron microscopy (SEM; JSM‐6510LV, JEOL Ltd., Tokyo, Japan) was performed on three remaining specimens from both the control (air abrasion only) and primer‐treated groups to characterize surface morphology and identify topographical differences at 500× magnification.

For bonding with resin cement, the acrylic resin cement (Super‐Bond Universal Kit, Sun Medical Co., Ltd., Shiga, Japan) was prepared using the bulk‐mix technique according to the manufacturer’s instructions. In the Super‐Bond group, the resin cement was applied directly to the bonding surface. In the group treated with both M&C Primer and Super‐Bond, the resin cement was applied to the primer‐treated bonding surface. The fabricated cylindrical acrylic resin was positioned on top of the resin cement. A glass slab was then placed following the same bonding procedure described earlier.

Immediately after bonding, all specimens were immersed in distilled water and stored in a temperature‐controlled chamber maintained at 37 ± 1°C for 24 h. SBS testing was subsequently performed using a universal testing machine (Shimadzu AGS‐X, Shimadzu, Kyoto, Japan). A shear load was applied using a sharp‐edged blade positioned parallel to the bonding interface between the Co–Cr alloy and the chemically cured acrylic resin surface, using a crosshead speed of 0.5 mm/min, according to the ISO 10477 standard [20]. The maximum load at failure was recorded, and SBS was calculated in MPa by this formula: SBS (MPa) = force (N)/area (mm^2^).

After failure, the failure mode was examined and classified using a stereomicroscope (SZX7, Olympus Corp., Tokyo, Japan) at 10x magnification. The failure modes were classified into three categories: adhesive failure, defined as separation occurring exclusively at the interface between the alloy and the acrylic tooth; cohesive failure, occurring within the alloy or acrylic tooth material itself; and mixed failure, characterized by the presence of both adhesive and cohesive components. The failure patterns were recorded and quantified, and the results were expressed as percentages.

Statistical analyses were performed using SPSS Statistics Version 25.0 (SPSS for Windows, SPSS Inc., IL, USA). The normality of the data distribution was assessed using the Kolmogorov–Smirnov test. As the data were normally distributed, comparisons among groups were conducted using one‐way analysis of variance (ANOVA), followed by Tukey’s HSD post hoc test for multiple comparisons. All statistical tests were conducted at a 95% confidence level, with significance defined as *p* < 0.05.

## 3. Results

The mean and standard deviation values for the various surface treatments—including PMMA, PMMA combined with Clearfil Ceramic Primer Plus, PMMA combined with M&C Primer, Super‐Bond Universal, and Super‐Bond Universal with M&C Primer—are summarized in Table 1.

**Table 1:** Mean shear bond strengths (MPa) and standard deviation (SD).

Group	Surface treatment	Shear bond strengths (MPa) ± SD
1	PMMA (as a control)	3.07 ± 0.48^a^
2	PMMA + Clearfil Ceramic Primer Plus	6.24 ± 1.07^b^
3	PMMA + M&C Primer	5.29 ± 0.91^b^
4	Super‐Bond Universal	8.01 ± 1.05^c^
5	Super‐Bond Universal + M&C Primer	7.67 ± 0.96^c^

*Note:* Groups sharing the same superscript letters are not considered statistically significant (*p* > 0.05).

The Super‐Bond Universal group demonstrated the highest SBS, with a mean value of 8.01 ± 1.05 MPa, whereas the PMMA group recorded the lowest bond strength at 3.07 ± 0.48 MPa. The mean SBS values (± standard deviation) for the other surface treatment groups were as follows: PMMA + Clearfil Ceramic Primer Plus, 6.24 ± 1.07 MPa; PMMA + M&C Primer, 5.29 ± 0.91 MPa; and Super‐Bond Universal combined with M&C Primer, 7.67 ± 0.96 MPa.

Statistical analysis using one‐way ANOVA revealed that the type of chemical surface treatment had a significant effect on SBS values (*p* < 0.001). Tukey’s HSD post hoc analysis showed that all experimental groups exhibited significantly higher SBS than the control group. However, no statistically significant difference was observed between the Clearfil Ceramic Primer Plus group and the M&C Primer group (*p* = 0.168), nor between the Super‐Bond Universal group and the Super‐Bond Universal combined with M&C Primer group (*p* = 0.919). Notably, both Super‐Bond Universal groups achieved significantly higher bond strength than all PMMA groups (*p* < 0.001).

**Figure 1:**
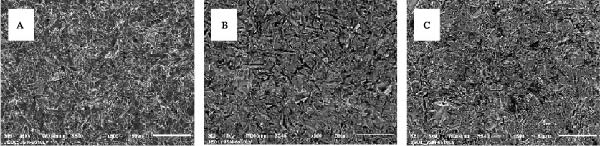
Representative scanning electron microscopy (SEM) images illustrating the surface morphology of treated Co–Cr alloy at 500× magnification: (A) air abrasion only, (B) air abrasion followed by Clearfil Ceramic Primer Plus application, and (C) air abrasion followed by M&C Primer application.

The SEM analysis (Figure 1) revealed that all treated Co–Cr alloy surfaces exhibited increased roughness and distinct microirregularities. The specimen subjected to air abrasion alone (Figure 1A) displayed a highly irregular and rugged surface morphology, characterized by sharp projections, deep grooves, and an uneven texture. In contrast, the surface treated with Clearfil Ceramic Primer Plus after air abrasion (Figure 1B) appeared more homogenized and slightly smoother. Although microstructural irregularities remained visible, they were partially masked by a thin coating, suggesting infiltration of the primer into the surface features. Similarly, the sample treated with M&C Primer (Figure 1C) exhibited a comparable surface morphology, with visible microgrooves partially covered by a uniform coating.

**Figure 2:**
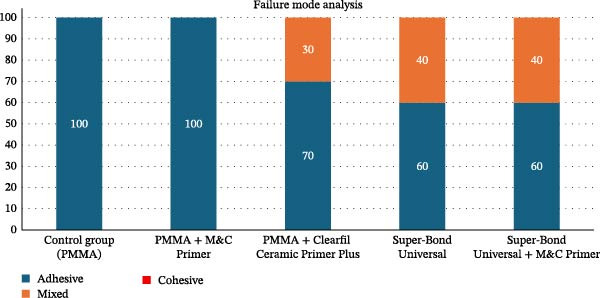
Bar chart, illustrating the distribution of failure modes observed after shear bond strength testing in all experimental groups. Each number in the relevant bar represents the percentage of failure mode.

**Figure 3:**
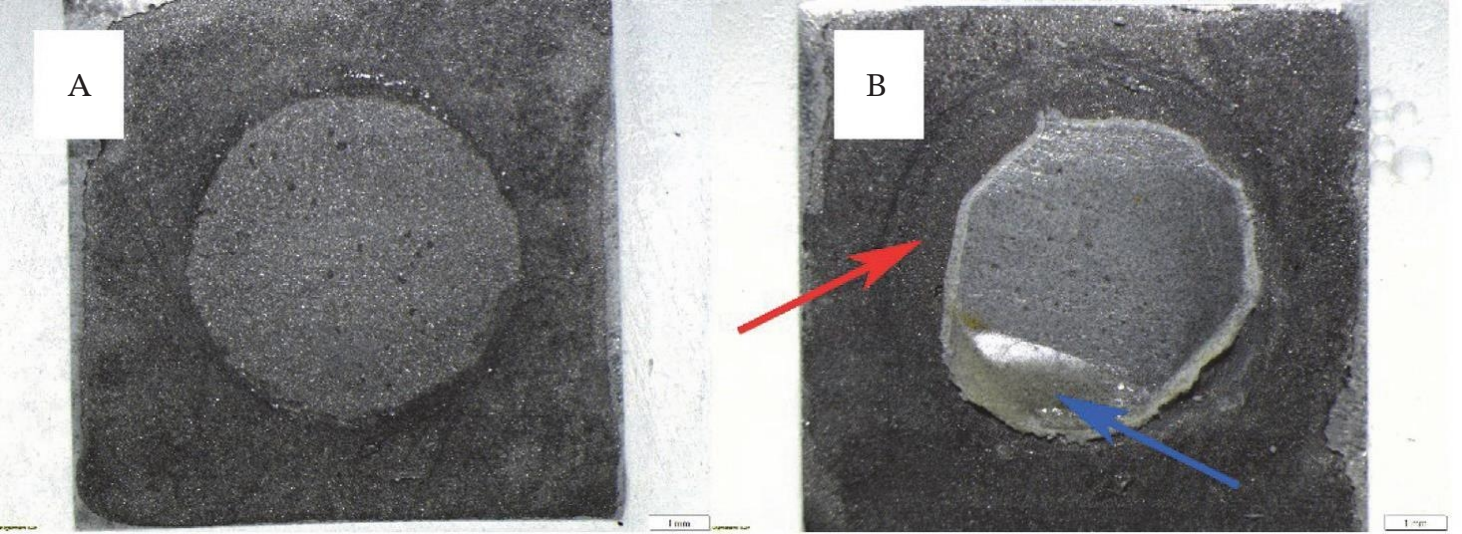
Representative fracture modes observed after the shear bond strength test under 10× stereomicroscope magnification: (A) adhesive failure and (B) mixed failure, characterized by cohesive fracture within the acrylic tooth (blue arrows) and adhesive failure at the interface (red arrows).

The distribution of failure modes across surface treatment groups is presented in Table 2 and Figure 2. In the control group and the PMMA with M&C Primer group, failures occurred exclusively at the adhesive interface. In contrast, the application of Clearfil Ceramic Primer Plus to PMMA resulted in a distribution of 70% adhesive and 30% mixed failures. The Super‐Bond Universal group and the Super‐Bond Universal group combined with M&C Primer demonstrated a distribution comprising 60% adhesive failures and 40% mixed failures. In addition, cohesive failure was not observed in any of the experimental groups. The fracture patterns of the specimens were classified and are illustrated in Figure 3.

**Table 2:** Distribution of failure modes observed after shear bond strength testing.

Group	Adhesive failure (%)	Mixed failure (%)	Cohesive failure (%)
Control group (PMMA)	100	0	0
PMMA + M&C Primer	100	0	0
PMMA + Clearfil Ceramic Primer Plus	70	30	0
Super‐Bond Universal	60	40	0
Super‐Bond Universal + M&C Primer	60	40	0

## 4. Discussion

Chemical surface treatments are essential for improving adhesion between Co–Cr alloys and acrylic resins. The present study demonstrated that surface conditioning protocols significantly influenced the SBS between Co–Cr alloy and acrylic resin teeth; therefore, the null hypothesis was rejected. Among the groups tested, Super‐Bond Universal exhibited the highest SBS, while the PMMA control group showed the lowest.

The superior performance of Super‐Bond Universal is attributed to the presence of 4‐META, a functional monomer known to enhance metal–resin adhesion [21, 22]. 4‐META interacts with the passive oxide layer on base metal alloys such as cobalt–chromium (Co‐Cr) and nickel‐chromium (Ni‐Cr) through its anhydride and methacrylate functional groups, forming stable chelation bonds and improving interfacial durability. Yoshida et al. [23] reported similar findings, noting that Super‐Bond produced the highest SBS among various primer–cement combinations.

The combination of Super‐Bond Universal and M&C Primer did not result in a statistically significant increase in bond strength compared to Super‐Bond Universal used independently. This may be due to the chemical redundancy of additional monomers, such as 10‐MDP and 3‐MPS, when 4‐META is already present. Koizumi et al. [24] similarly observed no difference in SBS between primer‐treated and untreated surfaces when using Super‐Bond Universal, further supporting this explanation. Additionally, tri‐*n*‐butylborane–initiated Super‐Bond demonstrated superior bond strength over benzoyl peroxide (BPO)‐initiated resins. These findings are in agreement with the present study, where the Super‐Bond group exhibited significantly higher bond strength than the primer‐treated group using PMMA containing BPO as the initiator.

PMMA combined with either Clearfil Ceramic Primer Plus or M&C Primer showed no significant difference in SBS. Both primers contain 10‐MDP and 3‐MPS, functional monomers that have been shown to enhance bonding by interacting with the oxide layer of Co–Cr alloys [25–28]. 10‐MDP forms ionic bonds with metal oxides via its phosphate group and polymerizes with methacrylate resins [29], while 3‐MPS contributes through siloxane linkages and copolymerization, collectively reinforcing the metal–resin interface [30].

The lowest SBS observed in the conventional PMMA group was likely due to the absence of functional monomers. Previous studies have confirmed that mechanical retention alone provides inferior bonding compared to combined mechanical and chemical treatments [31, 32]. Kalra et al. [19] reported that although airborne‐particle abrasion improved SBS, the addition of metal primers significantly enhanced the outcome.

Airborne‐particle abrasion using 50–250 µm Al_2_O_3_ particles is a widely adopted surface treatment technique to enhance micromechanical retention and increase surface energy at the metal interface [33]. This method removes surface oxides and contaminants while simultaneously creating microroughness that facilitates mechanical interlocking between the metal and the resin material. Several studies have validated that 50 µm Al_2_O_3_ significantly improves SBS, compared to untreated surfaces [12, 34, 35].

SEM analysis revealed that airborne‐particle abrasion without primer application produced a rough surface, which is conducive to micromechanical interlocking but lacks the chemical interaction necessary for durable bonding. In contrast, the application of Clearfil Ceramic Primer Plus and M&C Primer resulted in partial surface coverage, indicating the presence of chemical interactions facilitated by their functional components, such as 10‐MDP and 3‐MPS. These observations are consistent with previous studies demonstrating the beneficial effects of functional primers, particularly those containing 10‐MDP and 3‐MPS, in enhancing the bond strength between metal alloys and resin‐based materials [14, 36].

Failure mode analysis corroborated the bond strength results. The control and PMMA + M&C Primer groups demonstrated all adhesive failures, indicating poor interfacial bonding. In contrast, mixed failure patterns were observed in the Clearfil Ceramic Primer Plus, Super‐Bond Universal, and Super‐Bond Universal + M&C Primer groups, with up to 40% of specimens exhibiting mixed failure. These results are consistent with those of Tsuchimoto et al. [17], who also reported a correlation between higher SBS and the presence of mixed failure modes, reflecting improved stress distribution and interfacial integrity.

Clinically, a protocol involving air abrasion followed by bonding with a 4‐META‐based resin cement, such as Super‐Bond Universal, is recommended to achieve strong and durable adhesion in metal–acrylic prostheses. Functional monomer‐based primers, including Clearfil Ceramic Primer Plus and M&C Primer used with PMMA, also significantly improved bond strength compared to the untreated control group, supporting their use in situations where resin cement is unavailable. Reinforcing the metal–acrylic interface is particularly important in denture repair, where reliable bonding is essential for restoring function and prolonging the service life of the prosthesis.

The main limitation of this study is its in vitro nature. The experiments were conducted on laboratory‐fabricated specimens, which may not fully replicate clinical conditions. Therefore, future clinical studies are warranted to apply the proposed approaches to dentures used by patients, in order to confirm the clinical viability and practicality of these methods.

## 5. Conclusion

Within the limitations of this study, Super‐Bond Universal without primer demonstrated the highest bond strength when bonded to Co–Cr alloy and acrylic resin teeth. The subsequent application of functional monomer‐based primers, specifically 10‐MDP and 3‐MPS, prior to Super‐Bond Universal yielded comparable bond strength values. Notably, the application of primers before PMMA placement resulted in enhanced bond strength at the metal–resin interface.

## Funding

This research was supported by the Faculty of Dentistry, Srinakharinwirot University (Grant 404/2568).

## Conflicts of Interest

The authors declare no conflicts of interest.

## Data Availability

The data supporting the findings of this study can be obtained from the corresponding author upon reasonable request.
